# Acceptability and Feasibility of an Isometric Resistance Exercise Program for Abdominal Cancer Surgery: An Embedded Qualitative Study

**DOI:** 10.1177/1073274820950855

**Published:** 2020-10-09

**Authors:** Ferhana Hashem, David Stephensen, Amanda Bates, Tracy Pellatt-Higgins, Ralph (Nobby) Peter Hobbs, Malcolm Hopkins, Hazel Woodward, Charitini Stavropoulou, Ian L. Swaine, Haythem Ali

**Affiliations:** 1 Centre for Health Service Studies, University of Kent, Canterbury, Kent, United Kingdom; 2 Physiotherapy Department, East Kent Hospitals University Foundation NHS Trust, Kent and Canterbury Hospital, Canterbury, Kent, United Kingdom; 3 Haemophilia Centre, Royal London Hospital, United Kingdom; 4 Maidstone and Tunbridge Wells NHS Trust, Maidstone, Maidstone Hospital, Hermitage Lane, Maidstone, Kent, United Kingdom; 5 School of Health Sciences, City University, London, Northampton Square, United Kingdom; 6 Centre for Science and Medicine in Sport and Exercise, University of Greenwich, Gillingham, Chatham, United Kingdom

**Keywords:** abdominal cancer, surgery, rehabilitation, isometric resistance exercise, qualitative study

## Abstract

Although it is recognized in the early stages of cancer recovery that changes in lifestyle including increases in physical activity improves physical function, there are no clear findings whether low versus moderate intensity activity or home or gym exercise offer optimal benefit. Isometric-resistance exercises can be carried out with very little equipment and space and can be performed while patients are bed-bound in hospital or at home. This embedded qualitative study, based in an English hospital trust providing specialist cancer care, was undertaken as a component of a feasibility trial to evaluate the acceptability and feasibility of an isometric-resistance exercise program and explore the suitability of functional assessments by drawing from the experiences of abdominal cancer patients following surgery. Telephone interviews were undertaken with 7 participants in the intervention group, and 8 interviews with the usual care group (n = 15). The gender composition consisted of 11 females and 4 males. Participants’ ages ranged from 27 to 84 (M = 60.07, SD = 15.40). Interviews were conducted between August 2017 and May 2018, with audio files digitally recorded and data coded using thematic framework analysis. Our results show that blinding to intervention or usual care was a challenge, participants felt the intervention was safe and suitable aided by the assistance of a research nurse, yet, found the self-completion questionnaire tools hard to complete. Our study provides an insight of trial processes, participants’ adherence and completion of exercise interventions, and informs the design and conduct of larger RCTs based on the experiences of abdominal cancer surgery patients.

## Introduction

Surgery is one of the main types of treatment for abdominal cancer, with a high risk of post-operative complications and a notable decrease in physical function. Colorectal is the third most common cancer with 1.8 million cases worldwide in 2018 (10.6% of all cancers). Following colorectal cancer, the most common types of abdominal cancers also include stomach (6.1%), liver (5.0%), followed by cervical (3.3%), pancreatic (2.7%), kidney (2.4%) and ovarian (1.7%).^1^ Although it is recognized in the early stages of cancer recovery that changes in lifestyle including increasing physical activity can help to improve overall well-being, there are is very little clinical evidence in terms of how different modes of physical function and modes of exercise can be incorporated following active cancer treatment.^2,3^

Data from the United Kingdom suggests that a third of people living with and beyond cancer are completely inactive,^4-6^ and 20% reported moderate or severe difficulties with mobility or usual activities.^7,8^ The World Cancer Research Fund also found strong evidence that being physically active decreased the risk of one of the main types of abdominal cancer—that of colorectal cancer (WCRF Colon Cancer Report 2018). Evidence on physical activity for people recovering from cancer has been shown to improve physical function without increases in fatigue associated with exercise. However, there is little evidence to identify the optimum mode, frequency, intensity and duration of activity required for beneficial effects in cancer populations.^9^ In addition, there are no clear findings to compare whether low versus moderate intensity activity, or home-based versus gym exercise offer the optimal benefit. The rationale to determine whether home or gym exercises offer optimal benefit is based on previous trials evidence, which showed similar outcomes between patients regarding exercise maintenance and adherence.^10^ Isometric-exercises can be performed in a confined space such as in the home without requiring access to exercise equipment. Home-based isometric-resistance exercises therefore have the potential to have a positive effect on patients undergoing elective abdominal cancer surgery, as previous studies have shown that it can preserve and optimize their physical condition.^11^

While there is recognition of the benefits of aerobic physical activity,^12-15^ randomized or quasi-randomized studies with a clear resistence muscle strengthening component has seen low numbers of participants to allow for any statistically significant evidence to be used for or against this type of exercise program. The low numbers of participants has been due to recruitment taking place in single study centers with exclusion of participants with colon surgery. The methodological quality of these randomized studies are moderate, with unclear bias, difficulty in blinding trial participants and therapists, and in respect to description of the intervention, some information lacking in terms of equipment and methodology with regard to aerobic and functional activity components.^2^ Resistance exercise (or strength) training could help to facilitate recovery of muscle function.^16^ In particular isometric resistance (or static) training has been used in the rehabilitation of weak or atrophied muscle following surgery;^17^ important factors to be considered in establishing an effective training regime include the training intensity, the number and durations of voluntary contractions, and the number and frequency of training sessions.^17^ Isometric-resistance exercises can be carried out with very little equipment and space, and can be performed while patients are bed-bound in hospital or at home.^2^

The overarching aim of the **E**xercise **P**eri-**O**perative **P**rogramme (EPOP) trial was to develop an isometric resistance exercise program intended to improve the physical function of patients undergoing elective abdominal surgery for cancer, which aimed at expediting their return to normal physical function. The aims of the qualitative study were to qualitatively evaluate the acceptability and feasibility of an isometric resistance exercise program, and explore the suitability of assessments for physical function by drawing from the experiences of abdominal cancer surgery patients involved in the EPOP study.

Understanding the experiences of the patients is vital to consider which elements of the exercise program were relevant and useful, as well as which parts were less attractive. Exploring how different patients viewed and engaged with the intervention is helpful in ascertaining how it can be made more feasible to implement in the larger study.^18^

## Materials and Methods

### Overall Study Design

The study comprised 2 main phases. Phase 1 involved a systematic review followed by a development study aimed at designing, developing and refining the intervention with healthcare professionals and patients (further details have been described in a forthcoming publication). Phase 2 involved the delivery of the intervention with the intervention group receiving a 12 week isometric-resistance program (see Table 1). The isometric resistance exercise program consisted of a series of 10 static exercises, and utilized the abdominal, back, neck, arm, hand, leg and foot muscles. It was planned to be completed within a 12 week period following surgery. The usual care group were encouraged to walk when they felt able but did not receive physiotherapy advice on specific exercises. The qualitative study was a planned component of the trial and was incorporated as part of Phase 2 and comprised post-intervention follow-up exit interviews. The rationale of the qualitative study focused on assessing whether it was feasible to recruit patients with elective abdominal surgery, and second, exploring the adherence of patients to perform the exercises, finish the program and complete the functional assessments provided as questionnaires in the form of a booklet.

**Table 1.** Isometric-Resistance Exercise Program Developed for the EPOP Study.

	Name	Patient guidance
1	Abdominal muscles	*Lie on your back and bend your knees, keeping your feet flat on the floor. Bend your chin toward your chest, slowly lifting your head. Lower your head, keeping your chin as close to your chest as possible.*
2	Arms and Shoulders.	*Lie on your back with your arm by your side, elbow bent at a right angle close to your body. Hold your wrist with your other hand, across your body. Try to move your hand inwards while resisting the movement by pushing with your other hand. Hold for ___ seconds. Repeat twice with each arm.*
3	Trunk and Legs	*Lie on your back with your arms by your sides and your legs straight. Flex your feet toward you and press your knees down against the bed. Your ankles may raise off the ground. Hold for 10 seconds, then relax. Repeat twice.*
4	Hand and Arm	*Lie on your back with your arm by your side, elbow bent at a right angle close to your body. Hold your wrist with your other hand, across your body. Try to move your hand inwards while resisting the movement by pushing with your other hand. Hold for ___ seconds. Repeat twice with each arm.*
5	Foot and Lower Leg	*Sit with your legs stretched out in front. Put a looped exercise band around your feet. Turn the soles of your feet toward each other. Then turn the soles of your feet away from each other. Keep your knees straight, and facing the ceiling. Repeat twice.*
6	Abdominal muscles	*Lie on your back with your arms by your sides. Push your shoulders and heels toward the floor, lifting your pelvis off the floor. Hold for 10 seconds. Repeat twice.*
7	Arms and Shoulders	*Sit on a chair with your hands behind your neck. Place your elbows as close together in front of you as you can. Move your elbows apart as far as you can to the sides, then move them back together again as close as they will go. Repeat 5 times*
8	Trunk and Legs	*Sit on a chair, keeping your back straight. Lift your leg off the seat while keeping your knee bent, then return to the starting position. Repeat 5 times on each leg.*
9	Hand and Arm	*Stand or sit. Stretch one arm to the opposite shoulder, assisting the movement by pushing your elbow with your other hand. Hold the position for 10 seconds, then relax. Repeat twice on both sides.*
10	Foot and Lower Leg	*Lie on your back with one leg bent, foot flat on the floor, and the other leg straight. Flex your toes up to the ceiling and lift your straight leg up to about 20 cm off the bed. Make sure to keep your knee straight. Hold for 10 seconds. Repeat 5 times on both sides.*

The Phase 2 trial data collected included self-reported and objective measures of assessments. Self-reported physical function assessment measures were collected using the Short Physical Performance Battery Test (SPPB) and the “timed-up-and-go’ test (TUG). The outcome measures were collected at baseline, 2, 6 and 12 weeks. It also included the collection of qualitative data to capture patient experiences of the study. For the purposes of this paper, we report upon the findings from the embedded qualitative study in Phase 2.

### Participant Recruitment and Consenting Procedures

All patients were recruited from a large general hospital trust in England providing specialist cancer care who were undergoing laparotomies and laparoscopies for stomach, colorectal, and gynecological malignancies. Initially, potential participants were approached face-to-face by a member of their clinical team on behalf of the lead clinician by way of a signed letter, which was accompanied by a patient information sheet to assist the participant to make a decision whether to take part in the study. Participants provided written informed consent to take part in both the intervention and a follow-up telephone in”terview.

### Randomization

The trial was a 2-arm, parallel group trial with participants randomly allocated with a ratio of 1:1 prior to surgery. Randomization was performed using a secure, centralized and independent online randomization service (http://sealedenvelope.com/). The clinical trials administrator was issued with a password to access the service to randomize participants. Participants were randomized individually and in sequence within 24 hours of enrollment (i.e. screened as eligible and have provided informed consent). There was no participant allocation to specific surgical groups based on approach or primary diagnoses.

### Sample Selection

Following surgery after the 12-week intervention period had ended and all outcome measures and follow-up appointments had taken place, participants from both arms of the study were invited to take part in a telephone interview. Nineteen of the 23 participants were followed up for interview (the other 4 could not be called due to changes in contact details). Out of the 19 who were contacted by telephone, 4 did not participate in an interview: one participant was away on holiday; another wanted to wait until her chemotherapy had ended, and 2 did not respond to voice messages left to arrange interviews. In order to ensure the views of all participants were captured, purposive sampling techniques were employed to attain a maximum variation across the intervention and usual care groups, and to reflect the variation of the overall trial sample (n = 23). Participants were purposively selected to help construct a sample to achieve maximum variation, which included age and gender, date of surgery and the date of commencement and completion of the exercise program. Within a small sample, there were experiences where elements of the program were shared (Patton 2002, 2nd ed.), but there were also differences such as perceived baseline fitness, domestic environment and time commitments. From the exit interviews, it was not apparent whether the type of cancer influenced participants’ responses on performing the exercises or completing them.

From the embedded qualitative study, 7 interviews were undertaken with participants in the intervention group, and 8 interviews in the usual care group. The gender composition consisted of 11 females and 4 males. Participants’ ages ranged from 27 to 84 (M = 60.07, SD = 15.40). No males who were interviewed were allocated into the intervention arm, therefore no qualitative data relates to males undertaking the exercises. For the intervention itself, the sample consisted of 17 females and 6 males, which indicates that the participants invited for the embedded qualitative study was similar in terms of representative gender proportions (Table 2).

**Table 2.** Characteristics of the Patients of the Overall Trial Sample.

		Exercises plus usual care	Usual care
Males	N (%)	3 (27%)	3 (25%)
Females	N (%)	8 (73%)	9 (75%)
Age (years)	Mean (SD)	62.8 (15.5)	63.1 (13.9)
	Range	32-87	27-77
Height (cm)	Mean (SD)	164 (11.5)	167 (6.50)
	Range	149-185	157-175
Weight (Kg)	Mean (SD)	79.9 (21.9)	81.5 (18.9)
	Range	53-132	56.5-114
BMI	Mean (SD)	29.0 (4.57)	29.2 (5.40)
	Range	23-38.6	20.7-37.2
Type of operation	Open	4 (40%)	4 (33.3%)
	Laparoscopic	5 (50%)	7 (58.3%)
	Keyhole	1 (10%)	1 (8.3%)

### Data Collection

One off semi-structured telephone interviews were undertaken with patients to explore the experience of taking part in the EPOP feasibility trial (n = 15).^19,20^ Each interview lasted approximately 10 to 15 minutes. No other people were present during the interviews besides the participants and researcher. No other repeated interviews were carried out at a later time point. Interviews took place by appointment within 3 days to around 4 weeks following the end of the 12 week intervention. The topic guides were developed by the research team and piloted with the project’s Patient and Public Involvement (PPI) representatives (or lay members) who were abdominal cancer patient survivors. Two topic guides were developed—one for the intervention group and the other for the usual care group. The participants in the intervention group were asked about how they found the exercises, what they thought about the questionnaires, their thoughts about trial processes and their overall experiences of taking part in the study. The usual care group were asked the same question prompts but with omissions to questions relating directly to the exercise program and functional assessments. The interviewer used a topic guide to provide prompts for discussion, but participants were encouraged to take the conversation in directions they believed were important. All interviews were conducted between August 2017 and May 2018, with audio files being digitally recorded. Transcripts were not returned to participants for comment but were available on request if the participant wished to view it. No further field-notes were made during or after the interviews. Every interview was conducted by FH (female) who is an experienced qualitative researcher (at the time was Research Fellow based at the University) with a 15 year research portfolio working on patient experiences in health and social care and was part of the trial team.

The research nurses informed the participants that a member of the trial team (FH) would contact them following the end of the study to explore their experiences and to discuss any problems they may have encountered. The participants were also told that the researcher was based at the University and wanted to hear their views before progressing to the larger trial. Despite limitations with conducting follow-up telephone interviews (discussed further below), participants were asked at the end of the conversation if they wanted to share any experiences that had not emerged during the discussion and participants took the opportunity to explore new themes that were not included on the interview guide. Data saturation was achieved when interview data had reached “information redundancy” as no new ideas or themes were being generated.^21^

### Data Analysis

Interview data was coded by FH using a thematic framework approach, which took part in 4 stages. 1) Familiarization involving data immersing, gaining an oversight of the content and identifying topics of interest; 2) constructing an initial thematic analysis involving refining and coding the data into nodes and sub-nodes; 3) reviewing data abstracts which involved organizing the data to identify any coherent data groups; and 4) summarizing and writing up the data using summaries for each group of data appearing under each theme.^22^ All analysis was derived from the data. Although participants did not provide input directly onto the findings, the lay representatives had the opportunity to discuss them during trial meetings. This analysis was aided by the use of a qualitative software analysis program (NVIVO Pro 12) (see Table 3).^22-24^

**Table 3.** EPOP Phase 2 exit interviews coding frame.

Name	Description
**Adherence** (parent node)	*Patients’ views performing the exercises and completing the programme*
**Advice and support** (parent node)	*Training requirements, information received on exercises, advice sought (face-to-face or telephone), reassurance when performing exercises at home*
Exercise advice from nurses (face-to-face or telephone) (child node)	*Patients’ views on the advice received from the nurses, confidence in advice given, feeling able to raise questions and returning to the nurses for remote advice*
Exercise information guidance (child node)	*Patients’ impressions of the induction to the programme, whether their questions were addressed during the session and views on the guidance booklet on the exercises*
**Appropriateness of programme** (parent node)	*How s exercises to help recovery*
Exercise programme (child node)	*How patients felt about performing the exercises - easy, moderate, hard*
Functional assessments (questionnaires) (child node)	*What patients thought about the questionnaires / booklets they were given to complete*
**Optimal timing and setting** (parent node)	*For commencement and continuation of the exercise programme*
Hospital and_or home (child node)	*Responses from patients of their preferred setting to commence the exercises*
Timing following surgery (child node)	*Patients’ views on when they felt able to commence the exercises*
**Other factors identified encouraging participation** (parent node)	*External factors such as domestic environment, motivations for maintaining fitness, having a recovery plan, feeling in control*
**Randomisation and blinding** (parent node)	*Control & usual care groups’ views on trial processes*
**Recruitment** (parent node)	*Who recruited them, what explanations were given to them to help decide whether or not to take part*
**Retention** (parent node)	*Issues around retention and views from intervention or control groups about dropping out / attrition*
**Safety and suitability of exercises** (parent node)	*Patients’ views on how exercises felt, impact of surgery, adverse reactions from cancer treatment, any injuries*

## Results

### Summary Findings of the Trial

We recruited 23 patients to our intervention study. Eleven patients were able to successfully complete the intervention program safely and easily. Twelve patients completed the control arm of the study. There were no adverse incidents related to the exercise program itself, which required withdrawal from the program. There were 6 incidents related to surgical complications. The intervention measured physical function using the Short Physical Performance Test Battery (SPPB) and the Timed-up-and-go (TUG) test (full results to be published on a forthcoming paper).

### Finding of Qualitative Study

**Figure 1.**
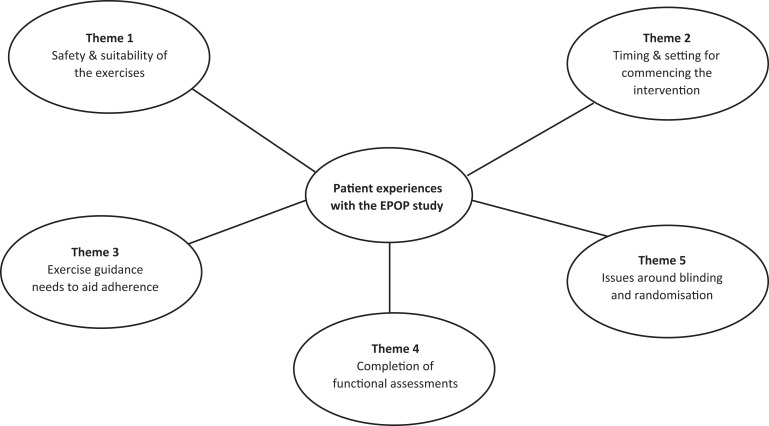
Themes regarding the experiences of patients on the EPOP isometric resistance exercise study.

The emergent themes that were identified from the qualitative interviews were: (i) safety and suitability of the exercise program; (ii) timing and setting for commencing the intervention; (iii) exercise guidance needs to aid adherence; (iv) completion of functional assessments; and lastly, (iv) issues around blinding and randomization (Figure 1).

### Intervention Acceptability

#### Safety and suitability of the exercise program

From the 7 participants who were allocated to the intervention group, their statements suggest that under supervision they felt the exercises were suitable and were safe for them to do. One participant mentioned that the exercises helped her build her strength and start to recover:

…so I did manage to get into them quite quickly and I continued them and I actually didn’t have a problem with those exercises at all and again I think, again it’s a positive thing you’re not just sitting there doing nothing, you’re thinking I’m actually helping myself to recover, to build my strength, so and you’ve got a lot of time during the day. You’ve got that time to

P09, female, intervention group

Another participant explained that the exercises were suitable and safe at the beginning, and did not have any concerns with them:

No, I just think generally everything…I think the exercises felt quite gentle up until the last…yeah…

P04, female, intervention group

One participant reported that she could not do some of the exercises due to having an open wound:

…well the problem I had which I don’t know whether [research nurse] actually explained to you, was that I had the wound that I had hadn’t healed properly…yes I couldn’t do the pelvic exercises…but the other, the top of body I could do.

P female, intervention group

She described in detail how the open wound impacted on her avoiding undertaking some of the exercises involving lying down and sitting up:

…oh yes, yes especially you know the upper body, I mean as I say with the pelvic area obviously I was a bit put back by that because obviously once I knew that I had a problem *with the stitching and I had, well I don’t know whether she’s put it down it was discharged and I had to have the wound opened and packed and everything, so obviously I didn’t want to put a strain on that so the exercises involved lying down and sitting up and all that I put on hold obviously because I didn’t want to put anything under strain. But the rest of them I found were fine.*

P10, female, intervention group

This participant’s statement indicates that having the exercises aimed at the lower body and pelvic area were not possible for her to complete given that she had an open wound that had not healed. She spoke about being able to put these specific exercises “on hold” so that she did not put a strain on that part of her body when performing the exercises, but at the same time, found it possible to be able to continue with the other exercises.

#### Timing and setting for commencing the intervention

The participants from the intervention group were asked if they found the exercises acceptable to carry them out after surgery. Some of the participants reported that they were able to complete the exercises and felt self-motivated to continue to do them after surgery, while others noted they did not want to commence them until after they arrived home. Participant P03 reported that she did not have any concerns about carrying out the exercises while still in hospital:

Yeah, fine. It was good. It felt nice, controlled and…you know one of the things you feel when you wake up from that sort of operation you realize you can’t go at things like a bull n a china shop anyway, it was nice to begin with something partly because it’s so very boring being in hospital anyway.

P03, female, intervention group

Another participant did not feel motivated to do the exercises until she came home:

I think it’s possibly a little bit unrealistic because you’re groggy from the effects of the anesthetic and you probably feel a little bit self conscious as well in hospital doing them, possibly. So I think really, realistically for me I didn’t start until I came back home, out again.

P04, female, intervention group

Yet, when she arrived home, once she started the exercises, she felt that she was making good progress to keep motivated:

…and maybe that’s because when you suddenly look at doing 30 and maybe it’s a bit of a mind-set that instead of thinking about doing thirty all in one go it’s that sort of mind-set of maybe the first sort of week

P04, female, intervention group

Another participant (P14, female, intervention group) mentioned that she found performing the exercises a little difficult, but she did find the time to do them on a regular basis:

…no no, I thought if it’s gonna—I didn’t expect it to be easy, that’s just you want to get better don’t you so you try and do everything you can really so, yeah…I tended to do them first thing in the morning and then some of them you had to do twice a day, I would find you know afternoon or something to do them…

P14, female, intervention group

### Appropriateness of Exercise Guidance & Functional Assessments

#### Exercise guidance needs to aid adherence

The participants were asked to comment on the instructions on performing the exercises. One participant (P14, Female, Intervention Group) struggled to understand the instructions on how to do the exercises:

…well I just couldn’t really understand it to be honest because you had to do, I had quite a few different ones to do and I couldn’t because of my shoulder I couldn’t do that and stuff so I tended to just do ones I could do and she narrowed it down so I was just sort of doing like 3 or 4 instead of well more

P14, female, intervention group

#### Completion of functional assessments

The participants who were interviewed in the intervention group had a variation of responses when commenting upon the functional assessments. Two respondents (P10 & P09) commented that the functional assessments were appropriate. One of the participants stated that:

…yeah no problem with those again, it’s quite nice because you can sort of get a feeling yourself of how you’re coming along…I could see the difference, then when I went back to the next one I could again tell the difference that I was stronger and I was able to do it better so it gave me a little bit of a measure myself.

P09, female, intervention group

There were 4 participants (P14, P03, P04 & P13) who struggled with and disliked completing the functional assessments. One participant (P13, Female, Intervention Group) commented that:

…mmm I don’t know, well I don’t know, I don’t think they did no…I tried to do my best I could and explained to [research nurse] cos I said you know I can’t keep up with filling these forms and she said “oh no” you need to tell them that.

P13, female, intervention group

Yet, another participant spoke about the functional assessments being a useful tool to measure progress by using them to self-reflect upon any improvements in her exercise performance. Once this participant had worked out how to record her responses, she thought the functional assessments were appropriate:

Yeah definitely, because when I looked at it I could see that, you know, it had gone from feeling hard…well not very hard but quite difficult to being actually this is quite easy. And that sort of fitted in with how my actual progress was going generally.

P04, female, intervention group

### Acceptability and Feasibility of Blinding and Randomization

#### Issues around blinding and randomization

Blinding was a challenge in the study, as the interview data suggests, the majority of participants were able to identify which group they were randomized into:

…well the ones that, well there were 2 groups weren’t there? I was the one that was doing the extra exercises—yes…

P10, female, intervention

Yeah, I had sort of said at the beginning I’d do it if I got allocated that and they said that you can’t chose so I was just lucky it came up but it’s what I wanted.

P03, female, intervention

It would appear from the quotations above that maintaining blinding was difficult in an exercise-related intervention. Out of the 15 participants who were interviewed (female = 11; male = 4), 11 participants commented that they knew which group they were randomized into, and 4 said that they did not know. Even though patients were given an explanation about being allocated into the usual care or intervention group, one respondent stated that he was still unaware that he was allocated into any specific group:

Interviewer: So do you know which group you were allocated to in the study?

Respondent: I don’t know that at all, even if I was allocated to a group.

P05, male, usual care

The participants were asked if they knew that by agreeing to take part in the study, this would involve being randomized and they accepted being allocated into groups:

I was hopeful that by being part of the [trial] group that I was going to be benefit from—or hopefully benefit from—the physio exercise program that…that would benefit me as well…Well obviously you have to have a control group…Well I mean I understood the concept of a control group in, you know, medical trials…so that was fine.

P01, male, usual care

…yeah it was the non-exercise…I didn’t mind because it was a random computer generated thing wasn’t it?

P08, female, usual care

The first male participant above explained that although he was disappointed not to be randomized into the intervention group, he understood that as he was taking part in a trial, he accepted the process of being allocated into the usual care group which he saw a part of trial logistical requirements. The second female participant also understood that she was randomized into the usual care group through a computer generated program that she also accepted was part of the trial process.

## Discussion

The EPOP feasibility study on isometric resistance exercise interventions following abdominal cancer surgery has helped to inform the design and conduct of the larger RCT by exploring the acceptability of the intervention and adherence to the program, safety and suitability, appropriateness of functional assessments, and trial processes around randomization and blinding. The feasibility of blinding in physical activity interventions, as demonstrated in previous studies, was shown to be a challenge as most participants were able to identify which group they had been allocated to. In addition, it was found that the functional assessments enabled participants to self-reflect to measure their own progress, yet, the self-completion questionnaire tools were reported to be hard to complete.

Participants who were in the intervention group had the ability and willingness to understand and adhere to the exercise program, as indicated by the 7 participants who were interviewed from this group. They commented about how they felt the intervention was safe and suitable, aided by the assistance of a research nurse, who was available to provide advice and help with adapting the exercises in relation to their post-surgery mobility requirements. Our findings concur with a study by de Almeida et. al (2017) of an early mobilization program in abdominal cancer patients after major surgery. The program based on core stability, aerobic and resistance training, orthostatic and gait training, was found to be safe and feasible.^25^ De Almeida notes the importance of a multi-professional approach including oncologists, surgeons, physiotherapists, nurses and psychiatrists, which contributed to high levels of adherence. The potential of a multi-professional approach in an exercise program is an important element to help with adaptations to the program and maintain adherence, and requires consideration for a future study.

One participant reported that due to an open wound she was unable to undertake any exercises in the pelvic area. Despite this, she continued with exercises to the upper body under clinical supervision. In a study by Schram et al. (2018) on an early mobilization program in colorectal surgery patients, it was found that patients were willing and capable of participating in a light to moderate intensity resistance-exercise program, by taking into consideration their specific post-operative status and stratified to reflect the patient’s individual needs.^26^ Although the patient in the EPOP study experienced mobility impediments due to an open wound, her response to continue with the exercises should also be noted, and that in discussion with the nurse she was able to carry on with clinical support. Malmstrom et al. (2013) found in their study on the long-term experiences of patients following esophageal or gastric cancer surgery, the changes in physical status resulted in patients feeling that they had lost control over their lives. Being involved in a supportive cancer recovery program allowed the patient in our study to take back control over her life.^27^ The importance of post-surgery cancer supportive care programs, at a wider level, demonstrates a need for patients to have active involvement in their recovery plans to enable them to feel in control of their lives.^28^

Participants commented that the functional assessments were useful for them to measure progress and set personal goals. Beck et al. (2020) found in their study on a prehabilitation intervention for patients preparing for abdominal cancer surgery showed that patients found it important to write the “dose” of exercise they had performed, which provided them a “personal” competition to be able to complete the activities and tick a box on a leaflet. The psychological impact of recording functional change in an important message from our study.^29^ However, it is also noted from the qualitative data collected from the EPOP study that the participants reported some common problems with completing the functional assessments, and highlights the need to explore reformatting tools in a far more “easy read” and accessible lay-out. Turnpenny et al. (2018) have noted that there are ways of adapting outcome measurement tools to make them more accessible through “easy read” materials, which are characterized by plain language, simple layout and format, and using images to illustrate key messages in the text.^30^ Although there are no common standards for producing “easy read” materials, there are national and international guidelines available to create accessible tools.^31,32^ The guidance will be consulted to re-design the functional assessments for the larger trial and any modified questionnaires will require re-validation.

The timing of the introduction of the exercise program was also a factor that may have impacted on execution and completion of the exercises. Although the evidence on the ideal time to promote physical activity is mixed,^33^ Shingler et al. (2017) noted in their study on prostate cancer survivors, that following surgery men in the study “felt able to embark on physical activity or nutrition interventions 6 weeks after…making this an acceptable timing for future interventions.”^34^ Shingler et al. stress the importance of identifying an optimum time to introduce lifestyle behavior changes for cancer survivors.^34^ In the EPOP study, drawing from the participants’ responses, some respondents noted that they would be able to start the exercises immediately after surgery while still in hospital, while other participants indicated not until they had been discharged home. The consideration of when to commence the exercise program is inter-linked with the issue of exercise setting and whether the setting encouraged patients to commence the exercises, or created a barrier to adherence. What this means for our study is that identifying an optimal time and setting for introducing exercise programs following surgery should not fit a “one size fits all” approach, but should be individually tailored should exercise support be offered. Karlsson (2019) suggests that exercise support should be considered with respect to an individual’s current physical activity and attitudes toward physical exercise.^35^

For the EPOP study, we found that the home-based setting of the intervention enabled the exercises to be carried out without any timetabling restrictions or transportation considerations, which the participants noted could be fitted in and around their daily routines. One of the key advantages of the intervention was that it was individualized rather than group-based which meant that participants were able to set personal goals for achievement, while noting benefits such as gains in mobility and strength, and recognizable improvements in physical performance.^36^ Further supervision and intermittent advice by way of a home visit or regular telephone call giving additional support could help identifying what is required for adherence, what the expected optimal achievement could be, as well as providing strategies to mitigate against some of the mobility issues encountered after cancer surgery. Therefore for a future trial, consideration of regular supervision with well-trained professionals could also enhance adherence, and improve participant experience.^37^

The lack of blinding noted in the responses from the participants in the EPOP study is a common challenge noted by El-Kotob and Giangregorio (2018) in RCTs of exercise, rehabilitation or physical activity interventions. They acknowledge that while a comparison or “usual care” group is an ideal to the intervention group, however, blinding in physical activity interventions are almost impossible resulting in the potential for bias. In our study, we found that the participants were at first attracted to taking part in the study as there was the potential to be in the exercise group. When finding out they were in the usual care group, they vocalized an element of disappointment. Although the EPOP participants declared feeling dissatisfied, this did not impact significantly on attrition or drop-out rates, unlike for example Barker et al.’s study (2016) whose participants indicated losing interest after being allocated to the control group and was the reason given for withdrawing from the study.^38^ Blinding in physical activity-based controlled studies has been shown to be a persistence problem, and requires further reflection for a future study.

The participants in the EPOP study found randomization an acceptable trial process, and acknowledged that they understood the logic of being allocated to either an intervention or usual care group. Unlike the Barker et al. study (2016), our participants had reconciled the fact that agreeing to taking part in the feasibility study would not necessary provide an opportunity to receive the exercise program. Considerations around determining the acceptance of randomization may be linked to the EPOP study having modest recruitment figures with 23 consenting in total, with 11 in the intervention and 12 in the usual care group, which enabled recruiters (research nurses) to offer an adequate explanation over what randomization involved.

We accept that there were certain study limitations. While we acknowledge the importance of capturing the views of the research nurses and physiotherapists who were involved in the delivery of the intervention, it was not possible to arrange these interviews due to time constraints and ongoing staff changes, which made it difficult to carry out this data collection. Future research might gain more insights into intervention delivery processes from the position of the healthcare professionals. Although the researcher was a member of the trial team, she did not meet the participants face-to-face who took part in the study, which may have impacted on being able to collect in-depth qualitative data. Although there were no explicit refusals by participants who were invited to take part in the qualitative interviews (n = 4), it would have been useful to identify whether these non-responders were unwilling or unable to take part. In addition, conducting interviews with those who refused to take part in the feasibility RCT would be insightful to understand whether changes in recruitment processes may be required for the larger RCT.

## Conclusion

In this embedded qualitative study, we examined abdominal cancer surgery patients’ perspectives of an isometric-resistance exercise program with respect to recruiting participants to an RCT, randomization and blinding, and exploring adherence of participants to perform and finish the program, and their experiences of completing the function assessments. Results indicated that participants had the ability and willingness to adhere to the program indicating they found it suitable and appropriate. One patient required supervised modifications to the program who experienced mobility impediments due to surgery. Some participants found the functional assessments difficult to complete with respondents declaring a distinct dislike of them. The timing and setting of the program was a factor that may have impacted on the commencement of exercises. The home-based setting alongside regular supervised advice was found to be very positive. The participants found randomization and recruitment an acceptable trial process.
